# Ziyin Bushen Decoction Alleviates Perimenopausal Syndrome in Rats by Enhancing Estradiol Production

**DOI:** 10.1155/2020/8895809

**Published:** 2020-11-07

**Authors:** Bi-Xin Tang, Qing-Yi Meng, Chan Xie, Shen-Shen Zhao, Kun-Lun Wu, Fang Wang, Le-Yi Du

**Affiliations:** ^1^Department of Traditional Chinese Medicine, Gongli Hospital of Shanghai Pudong New Area, Shanghai 200135, China; ^2^School of Traditional Chinese Medicine, Ningxia Medical University, Yinchuan 750004, China

## Abstract

Perimenopausal syndrome (PMS) has a high incidence rate and affects the physical and mental health of middle-aged and elderly women. The blockage of PMS is significant in improving the health of perimenopausal women. Currently, for PMS prevention and treatment, traditional Chinese medicine (TCM) has become an ideal choice because of its safety and effectiveness. This study aimed to explore the anti-PMS effects of Ziyin Bushen Decoction (DKTP) and the underlying mechanism. Thirty female Wistar rats were divided into 5 groups (*n* = 6): control group, low-dose DKTP group, medium-dose DKTP group, high-dose DKTP group, and nilestriol group. The estradiol (E2) level in rat peripheral blood was analyzed using an E2 Radioimmunoassay Kit, and uterine morphologic changes were examined by hematoxylin-eosin staining. Learning and memory ability of rats was assessed by Morris water maze (MWM) and novel object recognition (NOR) task. E2 synthesis, metabolism, and transport associated estrogen receptor-alpha (ER*α*), GnRHR, CYP17, CYP11A1, CYP19, 17*β*HSD, STS, and SHGB were assessed to explore the E2-promoting mechanism of DKTP during PMS treatment. The loss of learning and memory, the decreased estrous and uterine coefficient, and the presence of histopathological changes suggests a successful establishment of rat PMS model. Following DKTP or nilestriol treatment, the above results were reversed. E2 level in serum, uterine, and ovarian tissues was upregulated upon different concentrations of DKTP treatment, indicating that DKTP promotes the E2 level in a dose-dependent manner. DKTP also increased the expression of ER*α*, CYP17, CYP11A1, CYP19, 17*β*HSD, STS, and SHGB while decreased the GnRHR expression in uterine and ovarian tissues, revealing that these key molecules involved in estrogen synthesis, metabolism, and transport in PMS rats. We confirmed the anti-PMS effect of DKTP through enhancing E2 production. Exploring a novel drug based on improving E2 synthesis, metabolism, and transport may represent a novel strategy for PMS prevention and treatment.

## 1. Introduction

Perimenopause refers to a time period around menopause [1]. Perimenopausal syndrome (PMS) is the leading cause of various senile degenerative diseases with a high morbidity rate of 85% [2], resulting in an increase in the incidence of hypertension and coronary heart disease by 5–6 times, and thus greatly affects the physical and mental health of middle-aged and elderly women [3]. Therefore, effective prevention and treatment of PMS serve as a crucial role in protecting the physical and mental health of perimenopausal women.

In the pathological processes of PMS, multiple enzymes, including cytochrome P-450c17alpha (CYP17), cytochrome P450scc (CYP11A1), aromatase cytochrome P450 gene (CYP19), 17*β* hydroxy steroid dehydrogenase (17*β*HSD), and steroid sulfatase (STS), are all involved with the estrogen synthesis, metabolism, and transportation in ovarian granulosa cells. The gene expression and abnormal activity of these enzymes are the main factors contributing to the altered perimenopausal estrogen levels in PMS [4–6]. Estrogen is essential in regulating the functions of gonadal, neuroendocrine, skeletal, cardiovascular, and other tissues and cells, mainly exerting its biological effects by binding to estrogen receptors (ERs) in target cells [7]. Estradiol (E2), derived from follicle, corpus luteum, and placenta, is one of the most bioactive female estrogens [8]. There have been many research reported that the ovary function is gradually declining and the E2 secretion is decreasing in the perimenopausal women, causing follicular atresia and ovarian function decline, which forms the typical hormone level disorder. The dryness-heat, palpitation, insomnia, mood swings, and other clinical symptoms in perimenopausal women are directly related to the disorder of E2 levels, and the degree of disorder directly determines the severity of clinical symptoms [9, 10]. Therefore, finding safe and effective treatments to increase E2 levels may prevent and alleviate PMS.

Ziyin Bushen Decoction (DKTP) is a Chinese herbal formula used by Kunlun Wu, a famous Chinese medicine practitioner, and is principally composed of Liuwei Dihuang Pill (*Rehmanniae radix preparata, Fructus Corni, Moutan Cortex, Dioscorea opposita, Poria,* and *Alismatis Rhizoma*) [11] and Erzhi Pill (*Fructus ligustri lucidi* and *Yerbadetajo herb*) [12] with *Polygonatum*, *Cuscuta*, *Fructus Mori,* and *Pseudostellaria heterophylla*. DKTP can greatly improve the kidney yin deficiency of syndrome differentiation of symptoms such as hot flashes, night sweats, lassitude in loin and legs, sphoria with feverish sensation in chest, palms, and soles, dryness of throat, tinnitus, and insomnia. Previous studies have confirmed that DKTP increased serum E2 while decreased serum follicle-stimulating hormone (FSH) and luteinizing hormone (LH) [13, 14]. Current studies mainly focus on the effects of DKTP on the peripheral endocrine hormones of the hypothalamic-pituitary-ovary (HPO) axis, namely, gonadotropin-releasing hormone (GnRH); however, the anti-PMS effects of DKTP have not been reported [15, 16]. Therefore, further exploration of the mechanisms of the curative effect of DKTP in PMS can provide a better understanding towards the clinical practice of traditional Chinese medicine.

In this work, we identified the protective effect of DKTP in rat PMS model and explored the underlying molecular mechanism. Our data suggested E2 as a novel target for PMS intervention in the future.

## 2. Materials and Methods

### 2.1. Establishment of Rat PMS Model and Grouping

**Figure 1:**
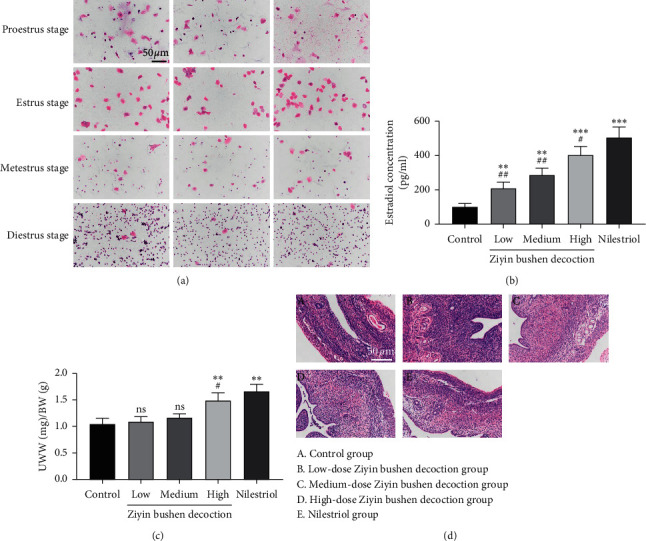
Effect of DKTP on uterus in perimenopausal rats: (a) exfoliative epithelia in vaginal smears of the female rats with regular 4-day estrus cycles by HE staining methods, displayed with a final magnification of 200x; (b) estradiol concentration; (c) the ratio of uterine wet weight to bodyweight (UWW/BW); (d) HE staining results of paraffin sections of rat uterus, displayed with a final magnification of 200x. ^*∗∗*^*p* < 0.01 and ^*∗∗∗*^*p* < 0.001, compared with the control group; ^#^*p* < 0.05 and ^##^*p* < 0.01, compared with the nilestriol group; ns, no significant difference.

Wistar female rats (220–240) were obtained from the Shanghai Lab, Animal Research Center, and adaptively fed for 7 days under standard laboratory conditions with a standard diet and water throughout the experiment. Rats in each group were anesthetized intraperitoneally with 75% ethanol, and the abdominal position was fixed. Under sterile operation, bilateral ovariectomy was performed. 80,000 U/penicillin was injected intramuscularly within 1 week after the operation. The vaginal cell smears were monitored for 5 consecutive days after surgery and checked if there was no estrous cycle which indicated the successful establishment of the model [17]. Cytological characteristics of vaginal exfoliation in different estrous cycles are shown in Table 1 and Figure 1(a). Animal experiments were performed in accordance with the Guide for the Care and Use of Laboratory Animals.

Thirty Wistar perimenopausal rats were randomly divided into 5 groups (*n* = 6): control group, low-dose DKTP group, medium-dose DKTP group, high-dose DKTP group, and nilestriol group. In the DKTP groups (low-dose, medium-dose, and high-dose), the rats were treated with DKTP pieces at 0.6, 1.2, and 2.4 g/ml in a volume of 10 ml/kg bodyweight, respectively. The nilestriol group was given 0.21 mg/kg bodyweight, a time/week. The suspension was made from distilled water with nilestriol tablets, dissolved in the required concentration, 10 ml/kg bodyweight, and administered by perfusion. The control group was given distilled water, 10 ml/kg bodyweight, once/day. Rats were administered by gavage daily for a total of 4 weeks. During treatment, rats in each group were weighed once a week and the dosage was adjusted according to their bodyweight.

### 2.2. E2 Content in Rat Peripheral Blood by Radioimmunoassay

The E2 level in rat peripheral blood of each group was analyzed using a E2 Radioimmunoassay Kit (BNIBT, China) following the manufacturer's instructions.

In brief, the kit included E2 standard preparations, 125I-E2, rabbit anti-E2 antiserum, QC serum, and donkey anti-rabbit separating agent. E2 and 125I-E2 were competitively bound to rabbit anti-E2 antiserum, which was detected by the donkey anti-rabbit separating agent. Therefore, a standard curve (*y* = 11675e-0.088x, *y*: E2 concentration; *x*: 125I-E2 binding rate) was determined by the kit. The E2 level in rat peripheral blood could be determined by detecting its binding rate to rabbit anti-E2 antiserum and applying the binding rate to the standard curve. The data are presented as the mean ± SD.

### 2.3. HE Staining

The tissues were fixed at 4% paraformaldehyde and then dehydrated, transparent, waxed, and embedded in paraffin. For histological examination, tissue samples were cut into 5 microns. After dewaxing and dehydration, tissue samples were stained with HE solution using a HE staining kit (G1120, Beijing Solarbio Science & Technology co., Ltd., China) for 5 min according to the manufacturer's protocol. Stained slides were analyzed by optical microscope under the same conditions. All slides were photographed at 200x magnification under a microscope.

### 2.4. Morris Water Maze Test

Morris water maze (MWM) test was used to evaluate rat spatial acquisition and memory retention. MWM consisted of a circular water pool (approximately 1.5 m in diameter and 0.8 m in height), which was divided into four quadrants. In the 4^th^ quadrant, a circular platform (10 cm in diameter) was placed 1 cm above the water surface. The pool was filled with appropriate water (23 ± 1°C). Rats were allowed to adapt to the environment before the experiment. Morris water maze test included place navigation performance (PNP) and spatial probe performance (SPP) analysis. In PNP part, the rats with swimming ability were placed in different quadrants in turn against the wall (all rats were required to move to the next quadrant) to undergo a swimming training 4 times/day in the 90 s. The trajectory and time for rat that boarded the platform area, named the escape latent period (ELP), were documented using an XR-XM101 recording analysis system. If the rat did not find the platform within 90 seconds, an experimenter introduced it into the platform, and ELP was prescribed as 90 s. After the completion of PNP part, the platform was removed, and SPP test was performed to assess the rats' ability to remember the spatial location of the platform. Rats were put into the water in the 4^th^ quadrant, and a typical track chart was made to assess the route for the rats crossing the platform within the 90 s.

### 2.5. Novel Object Recognition (NOR) Test

The rats were first placed in a cage for 5 ∼ 7 days for adaption. Three days before the training and testing, the rats were caressed for 1 min each day to eliminate the strangeness with the testers. And the rats were adapted to the arena (40 cm × 40 cm × 40 cm) 24 h before the training. At the training phase, two identical objects (O1) were placed, respectively, at the center of *A* and *B* areas in the neighboring quadrants. The rats were placed in the testing arena with their backs towards the objects. Ten minutes after the placement, the recording system was turned on to record the total time spent exploring each object. After 10 min of being placed in the testing arena, the rats were put back to the home cage. The testing phase was performed in the same testing arena as the training phase, but the object at the center of the *B* area was replaced by another object (O2). The rats were placed with their backs towards the objects as before. Ten minutes after the placement, the recording system was turned on to observe the exploring time *t*. After the testing, the rats were sent back to the original rear room.

### 2.6. Quantitative PCR Detecting System

Total RNA was isolated by the TRIzol method (Invitrogen, CA, USA), and cDNA was obtained by reverse transcription after DNA elimination. The prepared cDNA was amplified using SYBR Green PCR kit (Thermo, MA, USA), and the results were calculated by ABI Prism 7300 SDS Software. The primers used are as follows: CYP17A1-forward, 5′ TCACGATGAGAATGAATGGG 3′; CYP17A1-reverse, 5′ CAAGTAACTCTGCGTGGGTG 3′; CYP11A1-forward, 5′ TTCAACTTCCAGCCTCTC 3′; CYP11A1-reverse, 5′ AGACACCACCCTCAAATG 3′; CYP19A1-forward, 5′ ACTTCTAACACGCTCTTC 3′; CYP19A1-reverse, 5′ ACAGTCTTCCAGTTTCTC 3′; AKR1C15-forward, 5′ AGACAAGATGGCTGATGG 3′; AKR1C15-reverse, 5′ TGCGTCCTTACACTTCTC 3′; STS-forward, 5′ ACTGACAGGGTGATTGAC 3′; STS-reverse, 5′ TGGAGAAGCAGCCATTAG 3′; sex hormone-binding globulin (SHBG)-forward, 5′ CCTCACCAAAATCAGCAAAC 3′; SHBG-reverse, 5′ CCATCTCCCATCATTCAGC 3′; ESR1-forward, 5′ GAAATGGGCACTTCAGGAG 3′; ESR1-reverse, 5′ GCCAGGTTGGTCAATAAGC 3′; GNRHR-forward, 5′ ATAGGTCAGCAGCAGTAGC 3′; GNRHR-reverse, 5′ GGGATGTGTCGCAATGTAG 3′; GAPDH-forward, 5′ GGAGTCTACTGGCGTCTTCAC 3′; GAPDH-reverse, 5′ ATGAGCCCTTCCACGATGC 3′. GAPDH was included as an internal control.

### 2.7. Western Blot

RIPA lysis buffer (BYL40825, JRDUN Biotechnology, Shanghai, China) containing protease and phosphatase inhibitors was used to fully lyse the cells and tissues at 4°C. The amount of total proteins was quantified by the BCA method. Cell lysates were then subjected to 10% sodium dodecyl polyacrylamide gel electrophoresis (SDS-PAGE). After electrophoresis, proteins were transferred to the nitrocellulose membrane. The membrane was blocked with 5% nonfat milk (BYL40422, BD repackaging) at room temperature for 1 h and then incubated with the following primary antibodies: anti-ER*α* (1 : 300, Ab3575, Abcam), anti-CYP17 (1 : 2000, Ab125022, Abcam), anti-CYP11A1 (1 : 500, Ab175408, Abcam), anti-CYP19 (1 : 500, Ab18995, Abcam), anti-17*β*HSD (1 : 500, Ab167686, Abcam), anti-STS (1 : 1000, Ab34781, Abcam), anti-SHGB (1 : 300, 251544, Abbiotec), anti-GnRHR (1 : 500, Ab202848, Abcam), and anti-GAPDH (1 : 1000, #5174, CST). Subsequently, the membrane was washed and probed with an HRP-conjugated goat anti-rabbit secondary antibody (1 : 1000, A0208, Beyotime Biotechnology, China) for 1 h at room temperature. Blots were developed with an enhanced chemiluminescent (ECL) assay. Image *J* software was used to measure the relative grayscale according to the following formula: relative grayscale = (the grayscale of each protein−the grayscale of the background)/(the grayscale of GAPDH−the grayscale of the background).

### 2.8. Statistical Analysis

Data were expressed as mean ± SD. Statistical comparisons were made by one-way ANOVA and a post hoc test (LSD or Games–Howell) using GraphPad Prism 7.0 software. *p* < 0.05 was considered statistically significant.

## 3. Results

DKTP and nilestriol increased serum E2 concentration and improved rat uterus in PMS rats. Firstly, radioimmunoassay was performed to determine serum E2 changes in various groups. As shown in Figure 1(b), serum E2 level was low in control rats, which is consistent with the endocrine characteristic of perimenopausal rats. By contrast, DKTP significantly increased serum E2 level in a dose-dependent manner (*p* < 0.01 and *p* < 0.001), and nilestriol was more potent than DKTP in increasing the serum E2 level.

We then looked at the effect of DKTP in the uterine organ coefficient, which is calculated as the ratio of uterine wet weight (UWW) to bodyweight (BW). The UWW/BW in the nilestriol group and high-dose DKTP group was significantly higher than that in the control group (*p* < 0.01, Figure 1(c)), indicating that nilestriol and high-dose DKTP exerted nutritional and moisturizing effects on the uterus. In addition, the effect of the nilestriol is more potent than the high-dose DKTP (*p* < 0.05) and low or medium dose of DKTP had no significant effect on UWW/BW (*p* > 0.05) (Figure 1(c)).

Moreover, we examined the rat uterine structure by HE staining. In the control group, the uterine diameter and the lumen were narrow, the endometrium was thin, the number of glands was abundant, the blood vessels were rich, there were more fibrous connective tissue hyperplasia, and the lamina propria cells were dense (Figure 1(d), A). However, the uterine diameter of the DKTP and nilestriol-treated rats was enlarged and the endothelium was also increased. Among them, rats treated with nilestriol and DKTP at medium and high doses displayed a thicker endometrium, lighter staining, large glandular cavity, and secretion in the cavity. In addition, a large number of cytoplasmic secretory vesicles and loose connective tissue were observed in the uterine of nilestriol and DKTP-treated rats (Figures 1(d), B–1(d), E). These results suggested that DKTP treatment protected rats from uterine structure damage in PMS.

### 3.1. DKTP and Nilestriol Improved Rat Spatial Acquisition and Memory Retention

**Figure 2:**
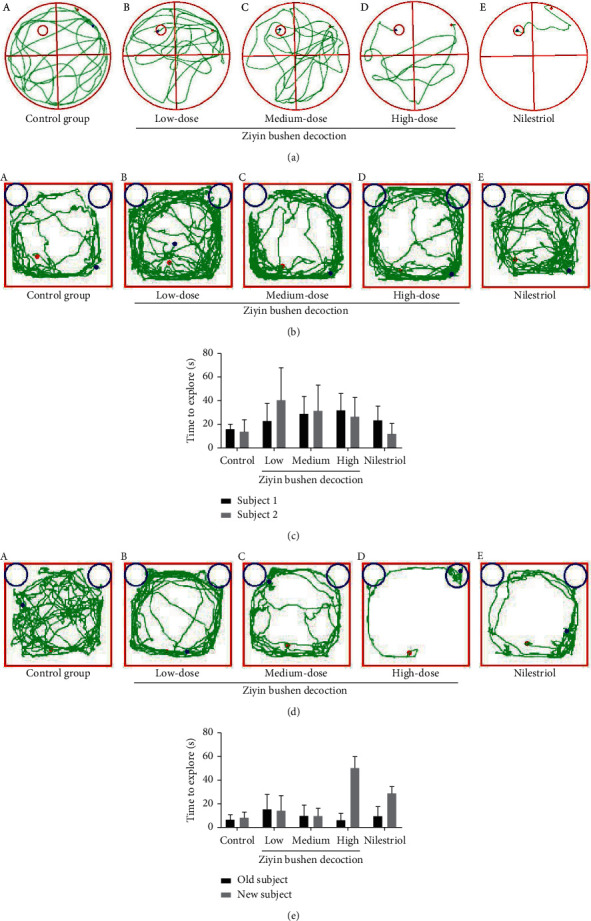
(a) The result of Morris water maze test; (b) the training phase in the novel object recognition test; (c) time for each group of rats to explore two objects; (d) the memory retention test in the novel object recognition test; (e) time for each group of rats to explore old/new objects. (a) Control group, (b–d) DKTP group (low, medium, and high doses), and (e) nilestriol group. ^*∗*^*p* < 0.05, compared with the time to explore old subject.

Rat spatial acquisition and retention were assessed using the MWM test. Our data showed that as the number of training days increased, the escape latency of rats looking for an underwater platform was generally shortened in all groups (Table 2). On the first day, there was no statistical difference in the escape latency of the rats in each group (*p* > 0.05), indicating that the starting point of learning in each group was basically at a comparable level. On the third and fourth days, the escape latency of the rats in the high-dose DKTP group and the nilestriol group was significantly lower than that in the control group (*p* < 0.05, *p* < 0.01, and *p* < 0.001). Moreover, the number of times and the platform residence time of the high-dose DKTP group and the nilestriol treatment group in the 90 s crossing the original platform were significantly higher than those in the control group (*p* < 0.05 and *p* < 0.01) (Table 3). The trajectories of the rats in the test group from the same quadrant to the water maze are shown in Figure 2(a). The results revealed that the swimming distance of the high-dose DKTP group and the nilestriol treatment group was significantly decreased, and the ability to find the platform was significantly improved than the rats in the control group.

### 3.2. DKTP and Nilestriol Ameliorate the Cognitive Impairment in PMS Rat

During the training phase, rats were allowed to freely explore objects for 10 min in the test box, and those with a total object exploration time of less than 20 s were excluded in the subsequent statistical analysis. The results showed that there was no significant difference in the time of exploration between the two subjects in the training phase (*p* > 0.05, Figures 2(b)–2(c)), suggesting that rats in each group had similar curiosity and motivation. Four hours after the end of the training, the memory retention test was performed. The rats in the high-dose DKTP group showed strong curiosity about the new objects and spent more time on new objects. Moreover, rats in high-dose DKTP group and nilestriol showed stronger recognition and memory ability for old objects that have been explored (*p* < 0.05, Figures 2(d), D and 2(e)). However, there was no significant difference in the exploration time of new/old subjects with the other groups (*p* > 0.05, Figures 2(d) and 2(e)).

### 3.3. Molecular Mechanism of DKTP in Promoting E2 in PMS Rats

**Figure 3:**
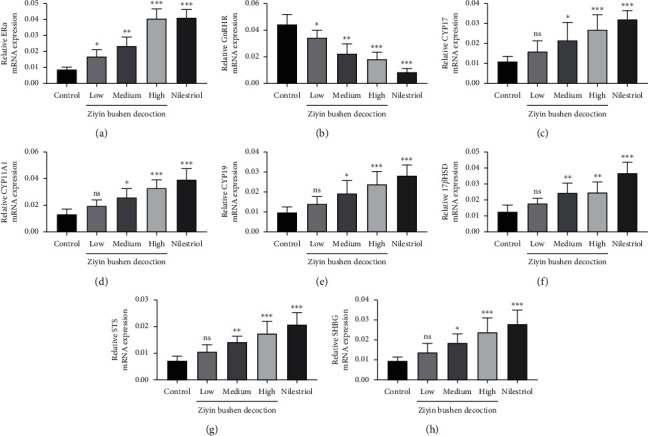
The Q-PCR results of ER*α* (a), GnRHR (b), CYP17 (c), CYP11A1 (d), CYP19 (e), 17*β*HSD (f), STS (g), and SHGB (h) in ovarian tissue. ^*∗*^*p* < 0.05, ^*∗∗*^*p* < 0.01, and ^*∗∗∗*^*p* < 0.001, compared with the control group; ns, no significant difference.

**Figure 4:**
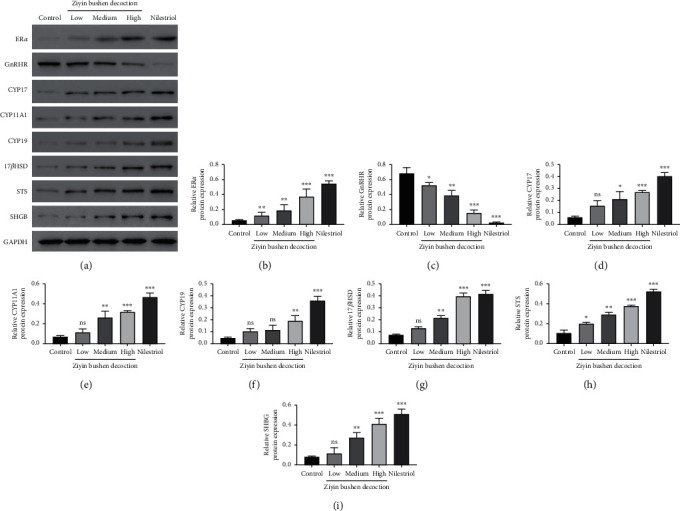
Western blot results (a) of ER*α* (b), GnRHR (c), CYP17 (d), CYP11A1 (e), CYP19 (f), 17*β*HSD (g), STS (h), and SHGB (i) in ovarian tissue. ^*∗*^*p* < 0.05, ^*∗∗*^*p* < 0.01, and ^*∗∗∗*^*p* < 0.001, compared with the control group; ns, no significant difference.

**Figure 5:**
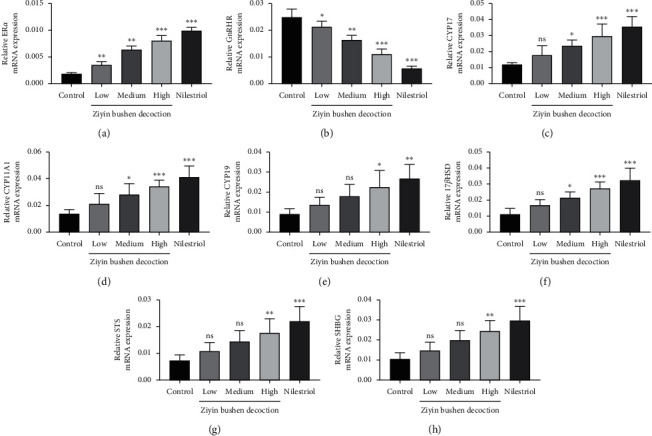
Q-PCR results of ER*α* (a), GnRHR (b), CYP17 (c), CYP11A1 (d), CYP19 (e), 17*β*HSD (f), STS (g), and SHGB (h) in uterine tissue. ^*∗*^*p* < 0.05, ^*∗∗*^*p* < 0.01, and ^*∗∗∗*^*p* < 0.001, compared with the control group; ns, no significant difference.

**Figure 6:**
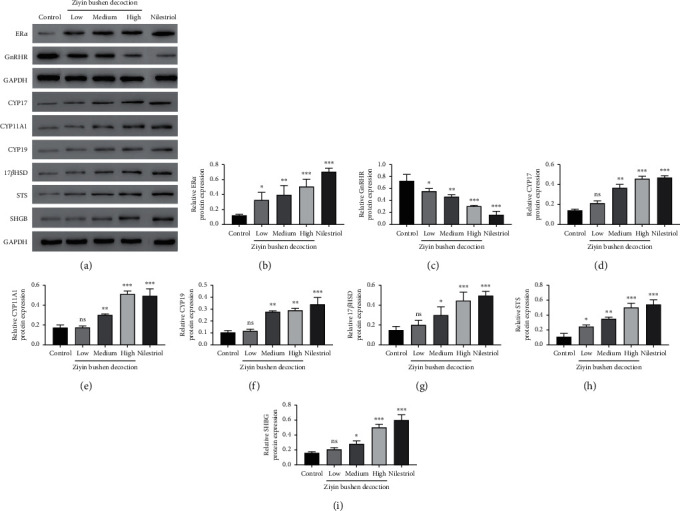
Western blot results (a) of ER*α* (b), GnRHR (c), CYP17 (d), CYP11A1 (e), CYP19 (f), 17*β*HSD (g), STS (h), and SHGB (i) in uterine tissue. ^*∗*^*p* < 0.05, ^*∗∗*^*p* < 0.01, and ^*∗∗∗*^*p* < 0.001, compared with the control group; ns, no significant difference.

To explore the molecular basis of DKTP effect, we evaluated the expressions of ER*α*, GnRHR, CYP17, CYP11A1, CYP19, 17*β*HSD, STS, and SHGB in ovarian tissues and uterine tissues by Q-PCR and Western blot (Figures 3–5). The results showed that DKTP significantly enhanced ER*α*, CYP17, CYP11A1, CYP19, 17*β*HSD, STS, and SHGB in a dose-depended manner when compared the control. The aforementioned effects of DKTP were similar to nilestriol (*p* < 0.05, Figures 3(a) and 4(b); *p* < 0.05 and *p* < 0.01, Figures 5(a) and 6(b)). In contrast, mRNA and protein levels of GnRHR were significantly reduced with the increasing concentrations of DKTP (*p* < 0.05, *p* < 0.01, and *p*< 0.001) and lowest in the nilestriol group (*p* < 0.001, Figures 3(b), 4(c), 5(b), and 6(c)). Therefore, DKTP treatment increases the expression of several key enzymes involved in estrogen synthesis, metabolism, and transport in PMS rats.

## 4. Discussion

In this study, we used a PMS rat model and explored the effects of DKTP on the pathological processes of PMS. The results showed that DKTP could significantly increase the E2 level in the serum of perimenopausal rats in a dose-dependent manner, and the UWW/BW of rats was significantly increased after high-dose DKTP and nilestriol treatment. Moreover, after DKTP treatment, rats' learning and memory abilities were enhanced, with improved cognitive impairment. DKTP induced increase in several estrogen-related key enzymes, and ER*α* expression indicated that this Chinese medicine could effectively alleviate PMS.

Perimenopausal women are often accompanied by obvious aging phenomena such as insomnia, forgetfulness, limb weakness, and lack of concentration in ovarian atrophy and endocrine disorders. One of the main manifestations of aging memory loss is learning and memory dysfunction. It has been reported in the literature that the method of Ziyin Bushen can improve the learning and memory ability of hyperlipemia vascular dementia rats [18]. Our research discussed for the first time the effects of DKTP on the learning and memory abilities of PMS rats, and the MWM's follow-up observation of the rat's motor exploration revealed that DKTP could improve the learning and memory ability of PMS rats to treat the perimenopausal neurodegenerative diseases and delay aging. Animals tend to explore new subjects. Rats with good learning and memory spend more time exploring new subjects in the environment, while the exploration time for the old subject is relatively reduced. Similarly, rats in the high-dose DKTP group showed significantly stronger recognition and memory ability for the old subject and strong curiosity to the new subject. These results indicated that memory impairment occurred in perimenopausal rats, and high concentrations of DKTP improved the memory of the rats, allowing them to remember old subjects, thereby distinguishing new objects and showing a strong tendency to explore.

The estrogen in normal menstrual women is mainly E2, which is derived from follicular intimal cells and follicular granulosa cells in the ovary, and plays a physiological role through interaction with estrogen receptor-alpha (ER*α*). ER*α* is a key factor in the estrogen effect, and its expression and activity has a crucial effect in regulating the neuroendocrine system. In addition, GnRH is a neurohormone necessary for maintaining the normal cycle, and it specifically binds to GnRHR [19]; it has been reported that E2 can downregulate the expression of GnRH and GnRHR mRNA [20]. Our results showed that all concentrations of DKTP significantly increased the mRNA and protein expression of ER*α* in the uterus and ovarian tissues while obviously decreased the mRNA and protein expression of GnRHR, suggesting that DKTP has a regulatory effect on estrogen levels and may improve the perimenopausal syndrome in rats by regulating GnRHR and promoting the self-stability of GnRH neuron.

Endogenous estrogen is mainly synthesized in follicles, and follicular cells are rich in CYP17 and CYP11A1. Under the promotion of LH, it can synthesize androgen from cholesterol through progesterone, the androgen produced enters ovarian granulosa cells, and the androgen is converted into estrogen by the action of the CYP19 [21]. In addition, 17*β*HSD completes the transformation of *E*_1_ and *E*2 into target cells [22], and STS also plays an important role in this process [23]. The gene expression level and activity of these enzymes have crucial effects on the level of estrogen in the body, thereby affecting the occurrence and treatment of perimenopausal syndrome. Besides, downregulation of perimenopausal ER*α* also affects the effect of estrogen, wherein reversibly binding to plasma transporter can greatly affect the biological activity and utility of estrogen, especially the important SHBG, whose expression level and degree of activation are important factors influencing the estrogen effect [24]. In this study, Q-PCR and Western blot were used to detect the mRNA and protein expression of estrogen synthesis, metabolism, transport, and other key enzymes and ER*α* in ovarian and uterine tissues. The results showed that the high concentration of DKTP could increase the mRNA and protein expression of these key enzymes to different degrees, and the high concentration of DKTP could achieve the same effect as nilestriol.

In this study, the DKTP treatment strategy is based on the Ziyin Bushen method, so the therapeutic effect was reliable. DKTP has an estrogen-like effect and regulates hormone levels in perimenopausal rats, and it can improve uterine atrophy in perimenopausal rats probably by exerting nutritional and moisturizing effects on the uterus. Moreover, the therapeutic efficacy of high dose of DKTP was comparable to that of the nilestriol group but was superior to nilestriol in improving memory. To our knowledge, this is the first study of the effects of DKTP on learning and memory in rats in PMS.

In conclusion, we identified DKTP as a potentially effective agent in improving perimenopausal symptoms and thus provide theoretical and clinical clues for the future prevention and treatment of perimenopausal.

## Figures and Tables

**Table 1:** Cytological characteristics of vaginal exfoliation in different estrous cycles.

Estrous cycles	Cytological characteristics
Prophase of estrous	Most of vaginal secretions were enlarged and slightly round with nuclear flattened epithelial cells, with occasional small number of acyclic keratinocytes
Estrous phase	The enlarged epithelial nucleus disappeared, became keratinized squamous desquamation, and accumulated to form a mass, most of which were keratinized cells, with some nuclear epithelial cells, but no white blood cells
Metestrum	There were nuclear epithelial cells, nucleated keratinocytes, and white blood cells
Diestrus	Most of the vaginal secretions are white blood cells with occasional small nuclei of flattened epithelial cells

**Table 2:** Results of positioning and navigation experiments of rats in each group ($\bar{x}$ ± *s*).

Groups	*N*	Escape latency (s)			
		1 d	2 d	3 d	4 d
Control	6	76.95 ± 25.42	64.2 ± 26.27	55.44 ± 25.48	54.55 ± 34.36
Low-dose	6	69.17 ± 19.61	60.35 ± 28.51	47.53 ± 35.39	36.41 ± 11.21
Medium-dose	6	65.39 ± 20.26	54.89 ± 30.58	31.79 ± 27.77	27.73 ± 27.08
High-dose	6	63.78 ± 23.06	37.39 ± 32.52	26.24 ± 28.64^*∗*^	10.18 ± 6.02^*∗∗∗*^
Nilestriol	6	64.32 ± 25.29	33.09 ± 21.38	20.33 ± 12.57^*∗∗*^	6.74 ± 5.56^*∗∗∗*^

**Table 3:** Results of space exploration experiments of rats in each group ($\bar{x}$ ± *s*).

Groups	*N*	Frequency of passes through the platform position	Platform dwell time (s)
Control	6	0.25 ± 0.52	1.72 ± 1.63
Low-dose	6	0.5 ± 0.58	2.47 ± 1.64
Medium-dose	6	1 ± 0.82	2.7 ± 1.51
High-dose	6	1.75 ± 0.51^*∗*^	2.86 ± 1.21^*∗∗*^
Nilestriol	6	3.5 ± 1.03^*∗∗*^	2.94 ± 0.73^*∗∗*^

## Data Availability

The data used to support the findings of this study are available from the corresponding author upon request.
